# Strategies for transferring resistance into wheat: from wide crosses to GM cassettes

**DOI:** 10.3389/fpls.2014.00692

**Published:** 2014-12-04

**Authors:** Brande B. H. Wulff, Matthew J. Moscou

**Affiliations:** ^1^Department of Crop Genetics, John Innes Centre, Norwich, Norfolk, UK; ^2^The Sainsbury Laboratory, Norwich, Norfolk, UK

**Keywords:** genetically modified, interspecific introgression, *R* gene stack, next-generation sequencing, cytogenetics

## Abstract

The domestication of wheat in the Fertile Crescent 10,000 years ago led to a genetic bottleneck. Modern agriculture has further narrowed the genetic base by introducing extreme levels of uniformity on a vast spatial and temporal scale. This reduction in genetic complexity renders the crop vulnerable to new and emerging pests and pathogens. The wild relatives of wheat represent an important source of genetic variation for disease resistance. For nearly a century farmers, breeders, and cytogeneticists have sought to access this variation for crop improvement. Several barriers restricting interspecies hybridization and introgression have been overcome, providing the opportunity to tap an extensive reservoir of genetic diversity. Resistance has been introgressed into wheat from at least 52 species from 13 genera, demonstrating the remarkable plasticity of the wheat genome and the importance of such natural variation in wheat breeding. Two main problems hinder the effective deployment of introgressed resistance genes for crop improvement: (1) the simultaneous introduction of genetically linked deleterious traits and (2) the rapid breakdown of resistance when deployed individually. In this review, we discuss how recent advances in molecular genomics are providing new opportunities to overcome these problems.

## INTRODUCTION

Modern agricultural systems apply an immense pressure on the plant immune system. The practice of planting only a few cultivars on a large swath of land creates an ideal landscape for the natural selection of pathogens that gain the ability to cause disease. The diversity within cultivated crops is often limited due to genetic bottlenecks that arose during domestication (Reif et al., 2005). Reduced genetic diversity is particularly attenuated in the context of disease resistance, as the evolution of the pathogen routinely outpaces plant breeding. In wheat (*Triticum aestivum* L.), cytogeneticists, breeders, and farmers have sought to overcome limited genetic diversity in disease resistance by identifying novel sources of resistance in the primary, secondary, and tertiary gene pools (Feuillet et al., 2008). Chromatin from over 52 species has been introgressed into wheat, demonstrating the remarkable plasticity of wheat and the importance of such natural variation in alien species for wheat improvement. From the seminal cytogenetic work of J. G. O’Mara, Ralph Riley, Ernie Sears, and others, the emphasis in wheat improvement through alien introgression was to identify sources of disease resistance. Research on this topic over the last 60 years has been reviewed in detail, including approaches used for introgression in wheat, the status of translocations within agriculture, and the importance of then novel technologies in the characterization of introgressions (Knott, 1987; Shepherd and Islam, 1988; Jiang et al., 1994; Friebe et al., 1996; Jauhar and Chibbar, 1999; Schneider et al., 2008).

In this review, we will describe briefly the history and biology of introgression in wheat, including how interspecific introgressions are made with wheat, the motivation for creating these introgressions, and how these introgressions have been used in agriculture. Our emphasis will be on discussing the fundamental change that next-generation technologies have had on accessing genetic variation from alien species. We will also highlight how novel approaches can be used to accelerate the breeding of disease resistance, and transform how we breed for resistance.

## THE HISTORY AND BIOLOGY OF ALIEN INTROGRESSION

### THE EVOLUTION OF WHEAT

Wheat is an allopolyploid formed through the sequential hybridization of three related grass species. Bowden created a classification system for individual genomes, which for bread wheat, included the A, B, and D genomes (Bowden, 1959). From extensive work on comparing the genomes of progenitor species and modern wheat, we know that the first hybridization event occurred approximately 0.8 Mya between *T. urartu* Tumanian ex Gandilyan (A genome progenitor) and an unidentified B genome progenitor that is related to *Aegilops speltoides* Tausch (Marcussen et al., 2014). The progeny of this hybridization event was the progenitor of pasta wheat (*T. turgidum* L. subsp. *durum* (Desf.) Husnot). Approximately 0.23–0.43 Mya, a hybridization event occurred between *T. turgidum* and the goatgrass *Ae. tauschii* Coss., whose progeny were the progenitor of modern-day bread wheat *T. aestivum* (Marcussen et al., 2014). This hybridization was recognized by humans ∼10,000 years ago and was subsequently selected and improved to generate modern bread wheat. The general capacity for grass species to undergo polyploidization has allowed for the generation of a number of synthetic allopolyploids, including triticale (*T. turgidum* × *Secale cereale* L.), tritordeum (*T. turgidum* × *Hordeum chilense* Roem. & Schult.), and secalotriticum (*T. aestivum* × *S. cereale*). Such hybridizations are evidence of the plasticity of grass genomes to accommodate higher ploidy levels.

### GENERATING ALIEN INTROGRESSIONS

**FIGURE 1:**
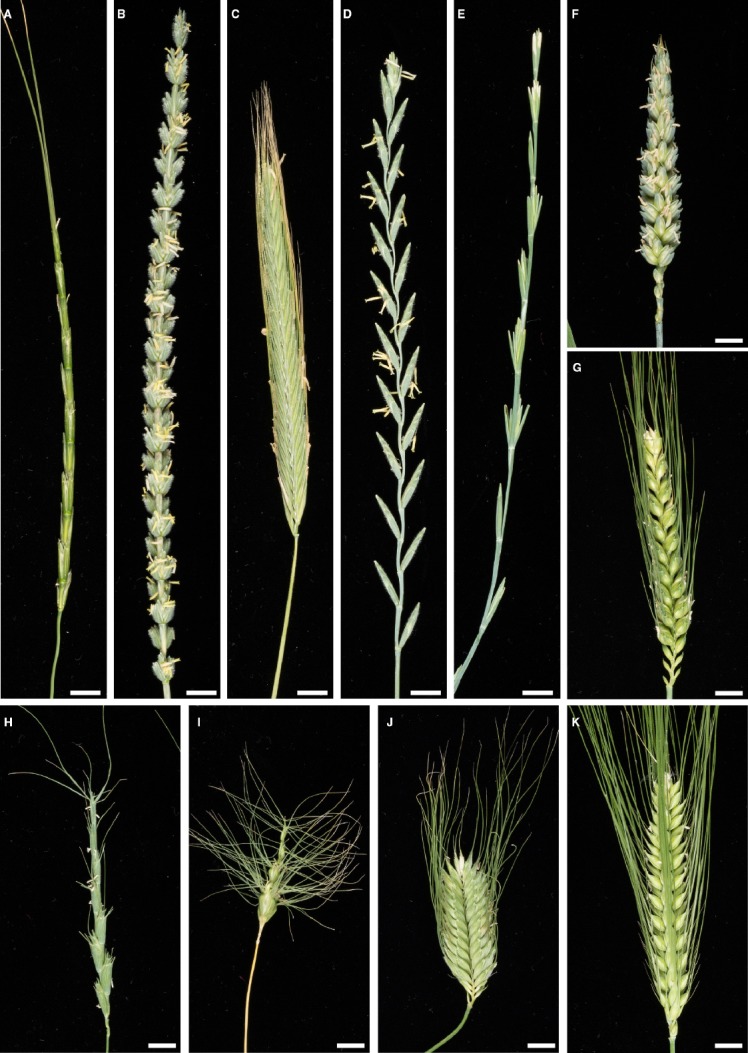
**Spike morphology of wheat and a selection of species used for introgression into wheat. (A)** *Aegilops markgrafii* (Greuter) Hammer, **(B)** *Ambylopyrum muticum* (Noiss.) Eig, **(C)** *Secale montanum* Guss., **(D)** *Thinopyrum intermedium* (Host) Barkworth & D. R. Dewey, **(E)** *Th. bessarabicum* (Savul. & Rayss) A. Love, **(F)** *Triticum aestivum* L. cv. Paragon, **(G)** *T. aestivum* L. cv. Highbury, **(H)** *Ae. speltoides* Tausch, **(I)** *Ae. umbellulata* Zhuk., **(J)** *T. timopheevii* (Zhuk.) Zhuk., **(K)** *Hordeum vulgare* L. Scale bar = 1 cm.

At the turn of the twentieth century cytogeneticists, breeders, and farmers began performing wide crosses between wheat and a number of grass species. This extensive search was motivated by a longing to recapitulate a number of desirable characters from alien species in wheat. While disease resistance was one of the most sought after traits, additional characters have included increased yield (Reynolds et al., 2001), early maturity (Koba et al., 1997), drought tolerance (Fatih, 1983; Molnár-Láng et al., 2014), salt tolerance (Forster et al., 1990; Hohmann et al., 1996), micronutrient efficiency (Schlegel et al., 1998), lodging resistance (Chen et al., 2011), cold tolerance (Rakesh and Sethi, 2000), and high protein content (De Pace et al., 2001). The search for genetic material that would improve these traits has included a diverse set of Triticeae species that comprise the primary, secondary, and tertiary gene pools of wheat. These gene pools include wild and cultivated species within the genera *Aegilops*, *Agropyron*, *Ambylopyrum*, *Dasypyrum*, *Elymus*, *Hordeum*, *Leymus*, *Lophopyrum*, *Psathyrostachys*, *Pseudoroegneria*, *Secale*, *Thinopyrum*, and *Triticum* (Figure 1).

Through attempts at transferring these traits into wheat, researchers encountered several barriers that impacted the possibility of introgression. These included a lack of chromosome pairing, preferential transmission of chromosomes harboring gametocidal genes, hybridization incompatibility due to sterility, and suppressed recombination, typically due to a lack of synteny. Several of these hurdles have been overcome through the treatment with chemicals for chromosome doubling (such as colchicine or caffeine), cold treatment, bridging crosses, mutation in chromosome pairing and gametocidal genes, and irradiation. These approaches will be discussed in more detail below.

Transfer of alien chromatin starts with an interspecific cross between wheat and the target alien species, which results in the generation of amphiploids. Amphiploids are hybrids that contain a diploid set of chromosomes from both parents; in this case this includes wheat and an alien species. Next, backcrossing is required to generate addition, substitution, translocation, and/or recombinant lines, the latter two events occurring either spontaneously, through DNA breaks, or via perturbation of the regulators of chromosome pairing. In normal circumstances, chromosome pairing occurs between homologous chromosomes and is tightly regulated by *Ph1* and *Ph2* (Riley and Chapman, 1958; Sears and Okamoto, 1958; Mello-Sampayo, 1971). Thus, in the wild-type background only homologous chromosomes recombine with one another. The development of mutations principally in *Ph1* heralded a new age for introgressions in wheat, as alien chromosomes can pair and recombine with the homoeologous wheat chromosomes in a *ph1* background (Riley and Chapman, 1958; Sears and Okamoto, 1958). Other approaches include the use of inhibitors of *Ph1* and *Ph2* derived from alien species, such as *Ph^I^* from *Ae. speltoides* (Chen et al., 1994).

Chromosome pairing requires that a sufficient degree of synteny exists. It is known that alien chromosomes may have substantial rearrangements relative to wheat (Devos et al., 1993), thus limiting the points at which homoeologous recombination can occur. Even in the event of proper pairing, several cases have proven that only a subset of chromosomes, arms, and segments can be introduced into wheat. This occurs as a result of deleterious allele combinations leading to necrosis or sterility, gametocidal loci, or non-compensating chromosomes. Such hybridization barriers often manifest at different stages in the development of introgressed material. In transferring the stem rust resistance (*R*) gene *Sr11* between wheat cultivars, Loegering and Sears (1963) identified the pollen-killer gene *ki* that distorted inheritance of *Sr11* (Loegering and Sears, 1963). In a distant cross, Islam et al. (1981) successfully generated disomic addition lines of all the barley (*H. vulgare* L.) chromosomes except 1H. The inability to introduce the entirety of barley chromosome 1H was due to the *Sterility in hybrids with wheat* (*Shw*) gene located on 1HL (Taketa et al., 2002). Preferential transmission contributes to the saturation of specific chromosomes and arms and not others (Miller, 1983; Qi et al., 2008). An extreme example of the preferential elimination of chromosomes is gametocidal activity located on chromosome 4S^*sh*^ from *Ae. sharonensis* Eig (Endo, 1990; Jiang et al., 1993). Lastly, non-compensating chromosomes can limit chromosome transfer, as was observed in the introgression of chromosome segments from *Leymus racemosus* (Lam.) Tzvelev (Chen et al., 2005).

Compatibility of species for the generation of interspecific hybrids and subsequent introgression seems limited only by the creativity and determination of researchers in applying novel techniques. Several approaches have been developed to access extremely wide crosses, including irradiation (Sears, 1956), tissue culture (Larkin and Scowcroft, 1981; Lapitan et al., 1984; Banks et al., 1995), and gametocidal genes (Tsujimoto and Noda, 1988). In many wide crosses, the F_1_ hybrid seed are shriveled and under normal conditions do not germinate. A simple major advance was the use of embryo rescue which has allowed greater access to a number of species in the tertiary gene pool (Sharma and Gill, 1983). Irradiation can be used to generate translocations, but these are often deleterious as a result of genetic imbalance. Sears observed this when he generated wheat–*Ae. umbellulata* Zhuk. translocations, wherein only one of 17 translocations was not deleterious (Sears, 1956). Use of irradiation also facilitated translocation of *Sr26* from *Lophopyrum elongatum* (Host) Love into wheat (Knott, 1987).

The size of an introgression can be highly variable. Size is dependent on compatible regions for recombination, as many alien chromosomes are rearranged relative to homologous chromosomes of wheat (Devos et al., 1993). Often the sites of wheat–alien recombination are unevenly distributed over the chromosome such that telomeric regions recombine more readily than pericentromeric regions (Curtis and Lukaszewski, 1991). It should be noted that such unequal rates of recombination are common within grass species, examples including *Brachypodium distachyon* (L.) P. Beauv. (The International Brachypodium Initiative, 2010), barley (Muñoz-Amatriaín et al., 2011), and wheat (Choulet et al., 2014). The most common form of translocations are those comprising entire chromosome arms. However, there are a few cases of cryptic introgressions, wherein an introgression cannot be detected using cytogenetic-based approaches. An excellent example of such a cryptic translocation includes the transfer to wheat of *Lr57* and *Yr40* from *Ae. geniculata* Roth (Kuraparthy et al., 2007b). Such observations demonstrate the limited resolution of cytogenetic-based approaches at resolving the presence of introgressions and the need for more sensitive assays. With the development of molecular markers, even small introgressions can now be identified (Dong et al., 2004; Kuraparthy et al., 2007a).

### TRANSFER OF RESISTANCE GENES INTO WHEAT

Disease resistance was a major focus in introgression breeding programs because it is a highly desirable agronomic character and an easily selectable phenotype. In addition, genetic-based disease resistance was the only component of crop protection before the development of modern fungicides. Early work by Hayes et al. (1920) and McFadden (1930) found they could successfully identify and introgress stem rust resistance from *T. turgidum* subsp. *durum* and *T. turgidum* subsp. *dicoccum* Schrank ex Schübler into bread wheat. Subsequent introgression into wheat over the next century would broaden and expand the repertoire of *R* genes functional against different pathogens of wheat including fungi, viruses, and pests (nematodes and insects). Importantly, wheat is host to a number of diseases such as barley yellow dwarf virus and eyespot, for which there is little or no known resistance that occurs within the species (Jones et al., 1995; Anderson et al., 1998). Thus, transfer from alien species becomes the only source of genetic resistance.

The continued search for novel sources of resistance derives from the desire to recapitulate the strong resistance observed in wild relatives and progenitors of wheat into the crop. Many studies have sought to catalog the number of genes that function in resistance within these inappropriate hosts against the pathogens of wheat. By adding the entire arsenal of individual chromosomes of rye to wheat, Riley and Macer (1966) established that at least three genes determine inappropriate host resistance in rye to wheat powdery mildew [*Blumeria graminis* (DC.) Speer f. sp. *tritici*] and two genes against wheat stripe rust (*Puccinia striiformis* Westend. f. sp. *tritici*). In contrast, they could clearly see that resistance to wheat stem rust (*Puccinia graminis* Pers. f. sp. *tritici*) from rye functioned poorly in wheat and resistance to wheat leaf rust (*Puccinia triticina* Erikss.) did not function at all. Not all resistance was qualitative, as resistance to take-all (*Gaeumannomyces graminis* var. *tritici* Walker) was distributed across most of the chromosomes of rye. Lastly, greater susceptibility to eyespot (*Oculimacula yallundae*; W-type) was observed with the addition of several rye chromosomes. Further work in a number of alien species has found that resistance can either be conditioned by a single locus or multiple loci, the latter of which likely contribute to the durable inappropriate host resistance observed in these species to wheat pathogens (Riley and Macer, 1966; Chen et al., 2005).

The polyploid status of wheat has a dramatic impact on the expression of resistance. Kerber and Dyck (1973) introgressed stem rust resistance from the einkorn wheat (*T. monococcum* L.) into durum and bread wheat. In the process they observed a progressive reduction in expression of resistance, from a relatively green leaf with chlorotic flecks at infection sites in the progenitor to infection sites with small or medium sized uredinia (type 1+ and 2) when present in tetraploid and hexaploid wheat, respectively (Kerber and Dyck, 1973). The nature of the wheat genome appears to be a double-edged sword: while polyploidy improves the expression of several agronomic traits, it comes at the expense of suppressing resistance through the negative interaction of homoeologous and non-homoeologous loci between genomes. The effect is systemic in wheat and examples include the suppression of *Pm8* by the *Pm3* locus (McIntosh et al., 2011; Hurni et al., 2014) and a widely conserved gene on chromosome 7DL that suppresses stem rust resistance in hexaploid wheat (Kerber, 1991).

## THE DISCOVERY AND DEPLOYMENT OF *R* GENES IN AGRICULTURE

While there are many obstacles that must be overcome for an introgression to be deployed in agriculture, several historical and contemporary introgressions have had or continue to have a significant impact on agriculture and food security. The most well known introgression is the rye (*S. cereale*) 1RS translocation that harbors genes involved in multiple disease resistance (*Pm8*/*Sr31*/*Lr26*/*Yr9*; Mago et al., 2005) and improved root structure (Sharma et al., 2011), as well as additional positive agronomic characteristics (Rajaram et al., 1983). Other historically and contemporary introgressions include the following resistances: *Sr36*/*Pm6* from *T. timopheevii* (Zhuk.) Zhuk., *Pm13* from *Ae. longissima* Schweinf. & Muschl., *Lr28* from *Ae. speltoides*, *Lr9* from *Ae. umbellulata*, *Pch1* and *Sr38*/*Lr37*/*Yr17* from *Ae. ventricosa* Tausch, *Gb2*/*Pm17* from *S. cereale*, and *Lr19*/*Sr25*, *Sr24*/*Lr24*, and *Sr26* from *L. elongatum* (Sears, 1956; McIntosh, 1991; Delibes et al., 1993; Friebe et al., 1996). Similar to the 1RS translocation, the translocation harboring *Lr19*/*Sr25* is associated with higher biomass (Sharma and Knott, 1966). The deployment of these genes has often been on a worldwide scale and has provided significant food security over the last century.

The limited set of introgressions that have made their way into agricultural systems is due to a number of hurdles faced after translocation of alien chromatin. Negative impacts on quality attributes include bread making quality in the rye translocation harboring *Sr50* (previously *SrR*; Mago et al., 2004), reduction in yield (The et al., 1988), the association of *Lr19*/*Sr25* from *L. elongatum* with yellow pigmentation in the flour (Autrique et al., 1995), or distorted inheritance patterns observed for *Sr43* (also from *L. elongatum*; Kibirige-Sebunya and Knott, 1983). In addition, while strong resistance may exist in an alien species, it may often be inaccessible for introgression due to cyclical translocations, fertility genes, or the presence of pairing genes such as *Ph1* (Jiang et al., 1994). Even if a translocation is compensatory with respect to fertility and other essential traits, there may be a potential tradeoff when chromosomes, arms, and segments are replaced. This can manifest itself by either directly impacting an agronomic trait or with the loss of a gene necessary for expression of a trait (Jiang et al., 1994). Often many introgressions are not studied in detail; so many beneficial and deleterious traits are likely undiscovered.

Agriculture faces a difficult challenge when trying to maintain genetic diversity in *R* genes. This is a result of the restrictions within agricultural systems (i.e., within university and commercial breeding programs) for selection of a number of traits; disease resistance is one component. Therefore, the rate by which agricultural systems can respond to changes in pathogen populations is limited by the diversity present within a breeding program and the method of selection. While a reservoir of resistance exists within wheat, high intensity farming coupled with the relatively short timescales of the evolution of the pathogen can lead to a relatively quick breakdown of resistance. The painstakingly slow process of plant breeding confounds the problem. Indeed, it “requires about the same length of time to develop a hybrid strain of wheat as it does to raise a boy” (Erickson, 1945).

In the case of the cereal rusts, single genes have been introduced onto wide swaths of land, often leading to a major breakdown in resistance, such as the defeat of the wheat stripe rust resistance gene *Yr9* on the rye translocation harboring *Sr31*. Such boom and bust cycles can be attributed to relying solely on a single *R* gene in a popular variety that is widely deployed. This imposes a strong selection pressure on the pathogen population to mutate the pathogen’s corresponding avirulence gene (Dodds and Rathjen, 2010). While these events are often pointed out as the inherent problem with the use of *R* genes in agriculture, there are also several examples of single genes from alien species that have been deployed and are durable, including *Sr31*, *Pm21*, and *Pch1*. At present, it is unknown why these genes are durable, and similarly, why other *R* genes are not (Ellis et al., 2014). Thus, we need to understand the basis for durability and identify novel strategies that effectively deploy *R* genes.

## NEXT GENERATION GENETICS—DISCOVERING, TRACKING, AND CLONING *R* GENES IN WHEAT

### THE WHEAT GENOME SEQUENCE

A good quality genome sequence is the cornerstone of exploring the relationship between genotype and phenotype. Obtaining the full genome sequence of bread wheat has been hampered by its colossal size. At 17 Gb, it is 40 times bigger than rice (0.43 Gb) and 126 times bigger than *Arabidopsis thaliana* (L.) Heynh. (0.135 Gb). In addition, the allohexaploid nature of the wheat genome, consisting of three highly related subgenomes derived from progenitors within the tribe Triticeae (Marcussen et al., 2014), has resulted in homoeologous genes with high sequence identity (Brenchley et al., 2012; International Wheat Genome Sequencing Consortium [IWGSC], 2014). To complicate matters further, ∼24% of the genes on all the chromosomes have undergone intrachromosomal duplications and the majority (81%) of the genome consists of repetitive DNA, primarily long terminal repeat (LTR) retrotransposons (IWGSC, 2014). On chromosome 3B, for which a physical sequence is available, the average distance between genes is 104 ± 190 kb (Choulet et al., 2014). These different layers of homoeologous and intrachromosomal duplication coupled with large tracts of repetitive DNA conspire to make bioinformatic assembly and scaffolding of wheat whole-genome shotgun (WGS) sequences highly challenging.

The first WGS sequence and assembly was published in 2012 by Brenchley et al. (2012). Based on an assembly derived from a 5 × coverage of 454 pyrosequencing of the cultivar Chinese Spring the authors identified 96,000 protein coding genes assigned across the A, B, and D genomes, and an estimated exome size of 170–340 Mb. This has now been substantially refined by the recent efforts of the International Wheat Genome Sequencing Consortium (IWGSC, 2014). In what was dubbed “slicing the bread wheat genome,” flow cytometry was performed on aneuploidy deletion lines of Chinese Spring to purify and Illumina-sequence individual chromosome arms thereby substantially reducing the complexity of assembling a highly redundant genome. This chromosome survey sequence identified 124,201 protein coding genes, 60% of which were genetically ordered along each chromosome based on a genetic map derived from low-pass sequencing of a digenic doubled haploid population (IWGSC, 2014). In a bid to produce a “gold standard” reference sequence, the IWGSC is currently generating and sequencing the minimal bacterial artificial chromosome (BAC) tiling path across individual chromosomes, a task recently achieved for the 1 Gb chromosome 3B (Choulet et al., 2014), the largest in the wheat genome. The target is to complete all 21 chromosomes by 2017 (http://www.wheatgenome.org/).

### REDUCING THE COMPLEXITY OF WHEAT

At 17 Gb, shotgun resequencing of the wheat genome still poses an economic barrier in most projects. Over the years various technologies have been developed to reduce the complexity of genomes before sequencing. Perhaps the simplest of these is to sequence the transcribed portion of the genome, known as RNASeq. In the case of hexaploid wheat this reduces the theoretical complexity by ∼50-fold. The reality is that a large fraction (50%) of wheat RNASeq reads correspond to a small fraction of the expressed genes (1–2.5%; Trick et al., 2012). Normalization can improve this (Zhulidov et al., 2004), but if a particular transcript is being pursued it has to be expressed in the tissue sampled. While useful as a marker discovery tool, RNAseq is not high-throughput in terms of genotyping large populations. In wheat, this niche is filled by high-density single nucleotide polymorphism (SNP) genotyping arrays, e.g., the 9K and 90K Illumina iSelect platforms (allowing up to 9000 and 90,000 markers; Cavanagh et al., 2013; Wang et al., 2014), and the recent Illumina Infinium platform allowing up to 1,000,000 markers. However, ascertainment bias can drastically reduce the effectiveness of this platform when genotyping alien chromatin that was not incorporated into the marker design process. Genotyping-by-sequencing (GBS), which typically interrogates toward 100,000 markers (Poland et al., 2012), is less biased than SNP-based arrays.

An altogether radically different approach is to sequence just the particular fraction of the genome likely to be associated with genetic variation impinging on the phenotype being studied. For example, the bread wheat exome constitutes only 1–2% of the total genome size. Specifically accessing this sequence space can be achieved by “exome capture.” Typically, short biotinylated RNA-baits complementary to the target sequence are hybridized to a next-generation sequencing (NGS) library, purified, and then removed by RNase digestion to leave a highly enriched target sequence. Exome capture tolerates a high mismatch—similar to the hybridization kinetics in a Southern blot—allowing capture of diverged sequences. Thus, individual probes in bait libraries can efficiently capture the homoeologous sequence space in tetraploid and hexaploid wheat (Saintenac et al., 2011; Henry et al., 2014). A yet smaller fraction of a plant exome includes the most common *R* gene encoded products: the nucleotide-binding leucine-rich repeat domain-containing (NB-LRR) proteins. A typical plant genome is populated by several hundred *R* genes of the *NB-LRR* class, many of which are clustered in complex arrays (Meyers et al., 2003). Recently, Jupe et al. (2013) developed an exome capture for the *NB-LRR* complement of potato. Their ensuing Resistance gene enrichment Sequencing (RenSeq) of the potato reference genome, *Solanum tuberosum* L. clone DM, allowed the discovery of 317 previously unannotated *NB-LRRs*. More than 50% of the reads were derived from *NB-LRRs*, corresponding to a 225-fold enrichment, with the identity between genomic *NB-LRR* and RNA bait ranging from 80 to 100%. More importantly, RenSeq on bulks of resistant and susceptible progeny from two potato populations segregating for single *R* genes to *Phytophthora infestans* (Mont.) de Bary allowed identification of SNPs in *NB-LRRs* linked to resistance (Jupe et al., 2013).

### GENETIC STRUCTURING OF WHEAT GERMPLASM FOR GENE DISCOVERY

The last 100 years of wheat breeding and research have resulted in a veritable treasure trove of genetic resources, including natural and induced variation. In the quest to discover, track, and clone genes underpinning important agronomic traits, wheat breeders and researchers have structured their germplasm in a multitude of different ways. The generation of a biparental F_2_ mapping population, followed by phenotyping and genotyping permits the rough localization of a major effect locus. This is often the first step in the long and hard slog of gene cloning, which is followed by fine mapping the locus, generating a physical sequence spanning the genetic interval (e.g., from BAC clones), isolating a series of independent mutants, identifying candidate genes, sequencing the candidate genes in the mutants, identifying the gene, and, finally, confirming its nature by complementation.

Double haploid (DH), recombinant inbred line (RIL), and near isogenic line (NIL) populations derived from biparental crosses, and multiparent advanced generation intercross (MAGIC) populations (Huang et al., 2012a; Mackay et al., 2014), all lend themselves to dissecting quantitative trait loci (QTLs). NGS-enabled genotyping of such populations are increasing the resolution and speed with which QTLs can be mapped and cloned in polyploid wheat (Trick et al., 2012; Saintenac et al., 2013a).

High-throughput and ultradense NGS-genotyping will also assist in the generation of wheat–alien introgression lines (ILs) with high background isogenicity (Reynolds et al., 2012). The construction of ILs harboring discrete, defined chromosome segments from the wild species, ideally representing a tiling path across the whole genome and within an otherwise uniform genetic background will improve our ability to perform accurate phenotyping, mapping, and ultimately cloning and combining minor and major QTLs for disease resistance from wild and alien species (Zamir, 2001). As described above, in cases where the donor line is a non-host for the wheat-adapted pathogen, this will allow genetic dissection of non-host resistance.

The ability to clone genes from biparental and multiparental population structures is limited by two factors: marker availability and recombination rate. While NGS technologies can now overcome the problem of developing markers, such map-based approximation approaches can run into difficulty due to a lack of recombination. Indeed, huge swaths of grass genomes have suppressed recombination. One recent study in wheat found that all crossover events on chromosome 3B occurred in only 13% of the chromosome (Choulet et al., 2014), while in barley 50% of the recombination occurs in 5% of the genome (Künzel et al., 2000). Even where recombination does occur, it is usually uneven particularly between wheat and alien chromatin (Qi et al., 2007), and rarely is the resolution high enough to directly identify the gene/causative SNP.

Genome-wide association studies (GWAS) on large cultivar collections take advantage of historical recombination (linkage disequilibrium) and have been used to identify candidate genes in diploid crops such as rice (Huang et al., 2010, 2012b), maize (Poland et al., 2011; Tian et al., 2011), sorghum (Morris et al., 2013), barley (Cockram et al., 2010), and polyploid *Brassica napus* L. (Harper et al., 2012). Until recently, this approach was limited in wheat due to a paucity of genetically mapped markers—a gold standard wheat reference genome promises to increase the resolution of GWAS in wheat to the point of gene identification.

A gene cloning approach that can overcome the biological limitation of recombination and the technical limitation of marker saturation is “mutational genomics.” This strategy is based on mutagenesis, e.g., with ethyl methanesulfonate, phenotypic screening for mutants, and sequencing the whole (or selected part of the) genome of multiple independent mutants to identify candidate genes. The number of mutations per mutant genome is easily 1000-fold less than the number of SNPs between two cultivars of the same species, thus greatly increasing the specificity of this approach over conventional inter-cultivar crosses. Mutational genomics, and variations thereof, have been widely used to clone genes in the model plant *A. thaliana* (Austin et al., 2011) and more recently in rice (Abe et al., 2012; Fekih et al., 2013; Takagi et al., 2013) and barley (Mascher et al., 2014). Wheat exome capture and sequencing (Gardiner et al., 2014; Henry et al., 2014) will allow genes to be cloned in this way from wheat and its relatives. A potential limitation of this approach is the requirement to phenotype large numbers of mutant families to ensure the recovery of enough independent mutants.

## NEXT GENERATION DISEASE RESISTANCE BREEDING—GENETICALLY MODIFIED CASSETTES

As described above, introduction of disease resistance into wheat from alien species by interspecific hybridization followed by repeated backcrosses to the domesticated parent and selection for resistance and agronomic performance is a slow process. The sexual incompatibility barrier can now be overcome for many relatives of wheat, but many wide crosses represent difficult challenges, often requiring bridging crosses, or crosses to defined mutant stocks. Subsequent multiple successive backcrosses to the elite parent often fail to eliminate all undesirable traits coming from the wild, undomesticated relative due to close linkage, low levels of recombination between the wheat and alien chromatin, or complete inaccessibility (e.g., Knott, 1980; Dundas et al., 2007; Mago et al., 2009, 2013). When coupled with a desire to introduce multiple sources of resistance, the likelihood of any transfer into elite cultivars requires extensive effort. However, exactly this, i.e., the combination of different sources of genetic resistance with non-redundant modes of action, would be highly desirable as it may extend the durability of resistance to a given pathogen (McDonald and Linde, 2002).

In the case of the rusts of wheat, a strategy has been proposed that would combine broad-spectrum major dominant *R* genes, e.g., three for each of leaf, stripe, and stem rust, with non-race, non-species specific adult plant resistance (APR) genes (Ellis et al., 2014). This proposal solves many of the problems associated with conventional breeding approaches. First, problems associated with sexual incompatibility and linkage drag are completely removed via the delivery of these genes as transgenes in a single cassette (Dangl et al., 2013; Ellis et al., 2014). This has the advantage of permitting the incorporation of disease resistance genes from species that cannot be introgressed, such as non-host species. Second, a genetically modified (GM) cassette would also ensure that the genes do not segregate in breeding programs, resulting in single genes again being exposed to the pathogen. This would be equivalent to the set of resistance genes present on the 1BL:1RS wheat–rye translocation. Lastly, GM cassettes can be shuttled from one cultivar to another, allowing breeders to focus on other agronomic traits.

While conceptually compelling, several technical, biological, and societal issues impede the generation of GM cassettes. Principally, the transfer of large GM cassettes into the wheat genome has not been established. *Agrobacterium*-mediated transformation efficiencies rapidly decrease as the size of the T-DNA increases (Park et al., 2000). A typical *R* gene with its regulatory elements is between 7 and 9 kb (Periyannan et al., 2013; Saintenac et al., 2013b), while the APR gene *Lr34* with regulatory elements is ∼16 kb (Risk et al., 2012). Thus, an *R* gene cassette comprising nine rust *R* genes (three for each of leaf, stripe and stem rust) and two APRs would range in size from 95 to 113 kb. This would increase still further if the cassette contained *R* genes that require a partner, such as *Lr10* (Loutre et al., 2009). While the field of synthetic biology is well placed to engineer large and complex modules of this magnitude (Gibson et al., 2008; Weber et al., 2011; Werner et al., 2012), wheat transformation platforms will need to be improved to ensure their efficient delivery or their assembly in the wheat genome by sequential site-specific incorporation of smaller components (Yau et al., 2013). Alternatively, the sequential transformation of individual components followed by screening for events targeted to a non-recombining region of the genome, such as the 1BL:1RS wheat–rye translocation, would also ensure future co-segregation of the *R* genes.

Once a GM stack is transferred into wheat, the disease phenotype of the genes in the stack will be largely epistatic to one another when scored with the pathogen. To functionally test the genes present in a GM stack, robust and rapid assays will be required to probe the function of each individual component in the stack. In the case of major dominant race-specific *R* genes, this will rest on the identification of the matching effectors, while for APR genes biochemical assays may be required. Bacterial type-III secretion-mediated delivery of effectors (Upadhyaya et al., 2014) could be used to transiently probe *R* gene function. Additionally, the development of transgenic wheat tester lines, each expressing a different effector, may provide a more robust output based on the macroscopic phenotype of testcross progeny.

While GM cassettes have great potential, it is important to recognize that they are limited by the same biological restrictions as conventional breeding. Similar to observations made with introgressions in wheat, the first major concern is associated with *R* gene suppression. This phenomenon occurs frequently when *R* genes, in particular alleles or homoeologous alleles, are combined by breeding or as transgenes (McIntosh et al., 2011; Hurni et al., 2014; Stirnweis et al., 2014). In the case of certain combinations of the powdery mildew *Pm3* alleles, the inhibition is post-translational and appears to occur through the formation of an inactive heteromeric complex (Stirnweis et al., 2014). Therefore, downstream breeding programs could inadvertently knock out components in an *R* gene stack, and this would go undetected unless each component were to be functionally tested again before variety release. Secondly, the expression of some *R* genes effective against biotrophs can open the door to necrotrophs in what is known as an “inverse gene-for-gene interaction” (Oliver et al., 2012). For example, the wheat necrotroph *Stagonospora nodorum* delivers the proteinaceous effector ToxA into its host which is recognized by the NB-LRR TSN1 leading to cell death and further proliferation of the necrotroph (Oliver et al., 2012). Removal of the *NB-LRR*, e.g., by mutation, restores resistance (Faris et al., 2010). Another example of trade-off between resistance to biotrophs and increased susceptibility to necrotrophs concerns the recessive powdery mildew *R* gene *mlo*. This gene has been extensively used in the last 40 years to effectively control powdery mildew in barley. However, *mlo* increases susceptibility to the necrotroph *Ramularia collo-cygni*, and the widespread use of *mlo* has been linked with the concomitant emergence of Ramularia leaf spot as a major disease of barley (Brown and Makepeace, 2009; McGrann et al., 2014).

Ultimately, the use of GM cassettes will depend on two key factors. The first factor is societal acceptance of GM wheat. Such acceptance does not limit scientists from generating the technology, but it does restrict the impact of the technology on agricultural systems. The second factor depends on the agricultural system. In the developed world, farmers have access to a vast array of tools for integrated disease pest management. Thus, input costs and yield would be the ultimate requirements. In contrast, the developing world often has fewer options with respect to controlling disease, and therefore may desire a combination of sustainable durable crop protection, but with a concomitant yield increase.

## FUTURE PERSPECTIVE

The genetically anchored wheat chromosome survey sequence, combined with sequence complexity reduction tools (e.g., exome capture and ultradense genotyping) on various structured populations (e.g., MAGIC, tiling path IL, and mutant populations) will revolutionize our ability to discover, map, and clone genes in wheat. This will only improve as more chromosomes are sequenced to a gold quality standard. However, *R* genes are among the genes in plant genomes that display the highest rate of diversifying selection, resulting in large copy number and sequence variation between different accessions of the same species (Meyers et al., 2003). Therefore, the pursuit of an *R* gene by map-based approximation in a given accession will likely take the scientist into “*terra incognita*” during the fine mapping stage. Negotiating this terrain is no simple task. Wheat is no exception and contains ∼600 *NB-LRRs* (Bouktila et al., 2014; IWGSC, 2014), although this is most likely an underestimate because short read sequencing technologies lack the discriminatory power to assemble such large gene families. An accurate, high-throughput, and cost-efficient long-read technology is required to overcome this limitation. The PacBio circular consensus sequence (CCS) platform (Travers et al., 2010) offers the required accuracy (99%) and read length (≤∼2500 bp) to characterize complex multigene families (Larsen et al., 2014; Zhang et al., 2014). If it were combined with *NB-LRR* exome capture on structured populations this could potentially directly define candidate *NB-LRRs*, thus substantially reducing the time and cost associated with *R* gene cloning in wheat and its relatives. We are poised therefore to overcome an important technical hurdle in the engineering of GM resistance cassettes, namely that of reducing the time and cost required to clone *R* genes in complex grass genomes.

It is clearly no longer a question of “can we clone resistance genes from wheat” but rather, “which are the best genes to clone” and “how best to combine them for different agricultural settings”? The issues to consider here are manifold and beyond the scope of detailed investigation in this review. In general, the more durable and environmentally stable a resistance gene is likely to be, the better a target it represents. Agricultural durability is difficult to predict, but a key stepping-stone can be gained from anticipatory breeding experiments in which the pathogen is mutated and then applied to a wheat cultivar carrying a defined resistance gene. In the case of wheat stem rust, the degree to which a race mutates to overcome a defined *R* gene depends on the combination of the *R* gene and the race (Luig, 1978). Another issue to consider is the extent to which a gene is being deployed in agriculture (Johnson, 1984). The rationale is that genes that have not yet been exposed to the wheat-adapted pathogen on a large scale have yet to be selected against, thus attenuating the timelines associated with break down of resistance. In the future, a systems-based approach that incorporates information from pathogen sequencing, the global population dynamics of defined effectors, and worldwide sampling to monitor the spread of pathogens, will help make informed decisions on which *R* genes to clone and stack, and to tailor this to specific regions and types of agriculture.

In conclusion, the scene is now set for cloning a score of wheat *R* genes in the next 3–5 years—the first step in a long-term program to develop biotech-based breeding for disease resistance in wheat. In the meantime, the technologies are being rapidly developed which will allow the *in vitro* engineering, delivery into wheat, and functional verification of gene cassettes. On the eve of the 20th anniversary since the first plant *R* gene was cloned, we are positioning ourselves to turn a new and exciting page in the history of agriculture—that of GM *R* gene cassettes.

### Conflict of Interest Statement

The authors declare that the research was conducted in the absence of any commercial or financial relationships that could be construed as a potential conflict of interest.
